# Unmet healthcare needs of people with disabilities: evidence from the 2018–2021 Korean disability and life dynamics panel

**DOI:** 10.1186/s12889-025-23048-w

**Published:** 2025-07-02

**Authors:** Jun Su Park, Bomgyeol Kim, Soo Hyeok Choi, Vasuki Rajaguru, Tae Hyun Kim

**Affiliations:** 1 Department of Public Health, Graduate School, Yonsei University, Seoul, Republic of Korea; 2 College of Nursing, Mo-Im Kim Nursing Research Institute, Yonsei University, Seoul, Republic of Korea; 3 Department of Healthcare Management, Graduate School of Public Health, Yonsei University, 50-1 Yonsei-Ro, Seodaemun-Gu, Seoul, 03722 Republic of Korea

**Keywords:** Unmet needs, Disability, Health service use, Needs assessment, Financial burden, Behavior model

## Abstract

**Background:**

People with disabilities frequently have higher healthcare demands and unmet needs. This study investigated the unmet healthcare needs of people with disabilities in Korea, addressing the disparities across disability types.

**Methods:**

We analyzed the 2018–2021 Korean Disability and Life Dynamics Panel using the vulnerable population behavior model. The dependent variable was unmet healthcare needs among people with disabilities. Trends in unmet healthcare needs were assessed annually using frequency and percentage analyses, with statistical evaluation conducted via a trend test Multiple logistic regression analysis was conducted to identify factors associated with unmet healthcare needs.

**Results:**

Responses for each survey wave were as follows: 2018 (*n* = 6,121), 2019 (*n* = 5,527), 2020 (n = 5,259), and 2021 (*n* = 5,021). The proportion of unmet healthcare needs was 9.1% in 2018, decreasing to 5.8% in 2020, and slightly increasing to 6.0% by 2021(p for trend: *p* < 0.001). Reasons for the unmet healthcare needs of people with disabilities include a lack of money, difficulty moving, and a lack of a professional workforce. Among the reasons for unmet healthcare needs, “lack of money” significantly decreased from 70% to 60.8%. In contrast, “difficulty in moving” and “a lack of a professional workforce” increased significantly. Unmet healthcare needs were significantly higher among individuals with intellectual or autistic disabilities (aOR = 1.55, 95% CI = 1.11–2.16) and visual disabilities (aOR = 1.42, 95% CI = 1.11–1.80) compared to those with physical disabilities. People with disabilities who had a travel time of 30 min or more to a health facility were more likely to have unmet healthcare needs (aOR = 1.45, 95% CI = 1.28–4.64).

**Conclusions:**

People with disabilities’ unmet healthcare needs are primarily due to financial constraints and accessibility issues, such as travel time and mobility difficulties. Governments and policymakers must make efforts to reduce and prevent unmet healthcare needs among people with intellectual and visual disabilities.

**Supplementary Information:**

The online version contains supplementary material available at 10.1186/s12889-025-23048-w.

## Introduction

A total of 2.65 million people in Korea are estimated to have at least one disability, accounting for less than 5.2% of the total population [1] and posing a significant challenge to the healthcare system [2]. Compared to those without disabilities, people with disabilities experience greater general and specialist healthcare needs and may report more unmet needs for healthcare and rehabilitation [3]. People with disabilities are also likely to experience attitudinal, physical, and system-level barriers to accessing healthcare services [4, 5]. Significant disparities between socioeconomic status and unmet healthcare needs are also observed in most countries. Low-income individuals have more unmet healthcare needs than higher income individuals [6]. Despite comprising a substantial proportion of the population, people with disabilities are particularly vulnerable to precarious healthcare access due to ableist structures [7]. Therefore, access to health care services should be improved to eliminate health care disparities among people with disabilities [8].

Previous studies on disability-related health and healthcare disparities have primarily focused on comparisons between people with disabilities and people without disabilities, resulting in a lack of research on disparities among people with disabilities [9]. As people with disabilities are not a homogeneous group, research on disparities in healthcare access by disability type is crucial [10]. People with disabilities have greater needs and less access to healthcare services, resulting in poorer health status than those without disabilities [5, 6]. Specifically, people with different types of disabilities may encounter distinct barriers to accessing healthcare services [11]. Therefore, disability-type disparities in unmet healthcare service needs must be explored to promote equal access to healthcare services for people with disabilities. Despite the increase in the number of studies on unequal access to healthcare, previous studies on unmet healthcare needs for people with disabilities were limited to specific types of disabilities or diseases or focused on functional performance [2, 5, 9–11].

This study contributes to the literature on healthcare access among people with disabilities by presenting recent evidence from South Korea. We first estimated the prevalence of unmet healthcare needs among people with disabilities based on the 2018–2021 Disability and Life Dynamics Panel (DLDP) database. Next, we examined the differences in unmet healthcare needs according to the disability type. This study aims to identify and determine the factors associated with unmet healthcare needs to help policymakers and future researchers better support people with disabilities.

## Materials and methods

### Theoretical framework

This study used the Behavioral Model for Vulnerable Populations to examine the factors influencing people with disabilities’ access to or utilization of social services [12]. The model is an extended and modified version of Andersen’s Behavioral Model, widely employed in healthcare utilization research [13].

Andersen’s Behavioral Model comprises three categories: predisposing, enabling, and need factors. Predisposing factors include inherent characteristics such as age and sex, which, although they do not directly influence the desire to use healthcare services, remain crucial indicators. Enabling factors refer to economic and sociodemographic factors such as householder, marital status, education status, and monthly household income, which influence the ability to access healthcare services. Need factors are the physiological and psychological conditions connected to an individual’s level of disability or illness that directly affect the utilization of health services [14].

### Primary data source

This study utilized the Korean Korean Disability and Life Dynamics Panel (KDLDP), a nationally representative longitudinal panel survey designed to examine the dynamic aspects of the lives of people with disabilities [15]. The DLDP has been conducted annually by the Ministry of Health and Welfare of South Korea and the Korea Institute for Health and Social Affairs under the Korean Disability Welfare Act (national approved statistics number: 438,001) since 2018 [16]. The DLDP targets individuals with disabilities in South Korea who completed their disability registration between January 1, 2015, and December 31, 2017, excluding those residing in disability care facilities, along with their household members. The survey was designed to enhance the precision of national disability statistics while considering practical constraints such as time and budget limitations. To achieve this, a stratified double-sampling method was employed. In the first stage, a preliminary sample was selected at the eup, myeon, and dong (local administrative unit) level to ensure the inclusion of rare disability types and individuals under 18 years old. In the second stage, the final sample was drawn using stratified sampling based on disability type, severity, and sex. As a result of the panel construction process, a total of 6,121 individuals with disabilities were surveyed.

### Sample of the data analysis

The DLDP data from 2018 to 2021 (waves 1 to 4) were utilized. To systematically construct a longitudinal panel dataset, 6,121 individuals with disabilities who had completed their disability registration between 2015 and 2017 were selected as panel participants, along with their household members. In 2018 (Wave 1, Baseline Survey), 6,121 participants were included. The response rates for each subsequent wave were as follows: 5,527 participants (90.3%) in Wave 2 (2019), 5,259 participants (85.9%) in Wave 3 (2020), and 5,024 participants (82.08%) in Wave 4 (2021). The Korea Disabled People’s Development Institute (KDPDI) conducted standardized data collection and processing to ensure data quality. There were no missing data in the survey items, and thus, all available panel participants were included in the analysis.

### Ethical statement

The data used in this study are available from the Korea Disabled People’s Development Institute (KDPDI) upon request. This study utilized fully de-identified secondary data and was exempt from ethical review and approval in accordance with the Enforcement Rule of the Bioethics and Safety Act of the Republic of Korea (Article 13, Paragraph 1, Subparagraph 3).

All participants provided informed consent at the time of the raw data collection, and this study was conducted in compliance with the principles outlined in the Declaration of Helsinki.

## Variables

### Dependent variable

The dependent variable was unmet healthcare needs among people with disabilities. This encompasses the unmet healthcare needs of outpatients, inpatients, and emergency room services. Each participant answered questions like “Have you ever experienced any difficulties in visiting a hospital in the past year when you needed to see a doctor (including a dentist)?” The responses were either “Yes” or “No.” The proportion of respondents who answered “Yes” indicated that they had unmet healthcare needs in the previous year.

Furthermore, the survey questions included the following: What is the reason for not using healthcare facilities? The responses were ① lack of money, ② difficulty in moving, ③ lack of a professional workforce related to people with disabilities, ④ previous bad experiences, and ⑤ others (lack of information, rejection for no reason, lack of medical equipment, etc.).

### Independent variable

The following variables were derived per Andersen’s Behavioral Model for vulnerable populations:

## Predisposing factors

This study identified demographics, disability type, and severity as predisposing factors. Male and female participants were divided into the following age groups: < 20, 20–39, 40–59, and > 60 years. In the DLDP, disability type is self-reported by individuals with disabilities based on their disability registration card, which is officially issued under the Korean Disability Welfare Act [17]. The 15 disability categories presented in the DLDP are identical to those defined in the Korean Disability Welfare Act, and a detailed explanation of these categories is provided in the Supplementary Materials. In the DLDP, these 15 disability types are grouped into seven major disability categories. In this study, we redefined the classification by adding multiple disabilities to the existing seven categories, resulting in a total of eight disability types. Disability types were grouped into eight categories: physical disabilities, brain lesions, visual disabilities, auditory or linguistic disabilities, intellectual or autism spectrum disorder, mental health disabilities, facial or internal organ disabilities, and multiple disabilities. These categories align with the classification system used in the DLDP, which defines disability based on physician-diagnosed severity and functional limitations [16]. Disability severity was classified as mild or severe according to their disability grade.

### Enabling factors

The marital status was surveyed only for participants aged 19 and older. Participants aged < 19 years old were considered single. The marital status was classified as “Yes” (living with spouse) and “No” (bereavement, separation, divorce, and unmarried). Educational status was categorized as elementary school or below, middle school, high school, college or above. Householder status was redefined based on the individual's role within the household. If the person with a disability was the head of the household, they were categorized as yes. In Korean won (KRW), monthly household income was categorized as follows: < 1 million, > 1 million and < 2 million, > 2 million and < 3 million, > 3 million and < 4 million, and ≥ 4 million. Disability-related limitations in ADLs were calculated using a four-point Likert scale, averaging 14 daily living limitation questions. The ADL scale has been developed and its reliability (Cronbach’s alpha) verified in the Korean Disability and Life Dynamics Panel. The validity and reliability of the K-ADL (Cronbach's α = 0.937) have been previously confirmed [18]*.* The higher the score, the greater the limitations of daily life. Travel time to health facilities was divided into < 30 min and > 30 min.

### Need factors

The Short Form Center for Epidemiologic Studies Depression Scale (CESD-11-D) was used to measure depressive symptoms in people with disabilities [19]. The validity of the Korean version of the CESD-11-D for screening depression is well established. The procedures for translating and measuring these properties, including the reliability and validity of the Korean version of CES-D-11, have been comprehensively reported by Cho and Kim [20]. The authors employed a double-translation and back-translation process to produce the final version. The reliability of the final version, assessed using Cronbach’s α, was 0.893. Additionally, the authors found that the scale exhibited strong concurrent and discriminant validity.

Participants were asked to answer 11 questions concerning their depressive condition using a four-point Likert scale. This score ranges from 0 to 60, with high scores indicating a high severity of depression. We used a CESD-11-D cut-off score of 16. Self-rated health status was divided into good and poor, and chronic diseases were classified as absent or present.

### Statistical analysis

The data analysis was performed in three steps. First, the study estimated the trends in unmet healthcare needs per year using frequency and percentage analyses. To statistically evaluate these trends, a trend test (*p* for trend) was conducted. Second, the Lao-Scott chi-square test and t-test were performed to calculate the association between the unmet healthcare needs of people with disabilities and the variables. According to people with disabilities’ predisposing, enabling, and need factors, covariates were also reported as frequency and percentage for categorical variables and mean and standard deviation for continuous variables. Finally, a multiple logistic regression analysis was conducted using a generalized estimating equation (GEE) model to identify factors associated with unmet healthcare needs. We used a binomial distribution and a logit link function for the binary outcome variables. The temporal variable was the survey wave (every year), and person identification was used to identify repeated subjects using an unstructured working correlation matrix for the GEE model [21, 22]. The results are presented as Adjusted odds ratios (aOR) and 95% confidence intervals (CI). All calculated values were two-sided, and statistical significance was set at *p* < 0.05. All statistical analyses were performed using SAS software (version 9.4; SAS Institute, Cary, NC, USA).

## Results

### Annual trends in reasons for unmet healthcare needs

**Fig. 1:**
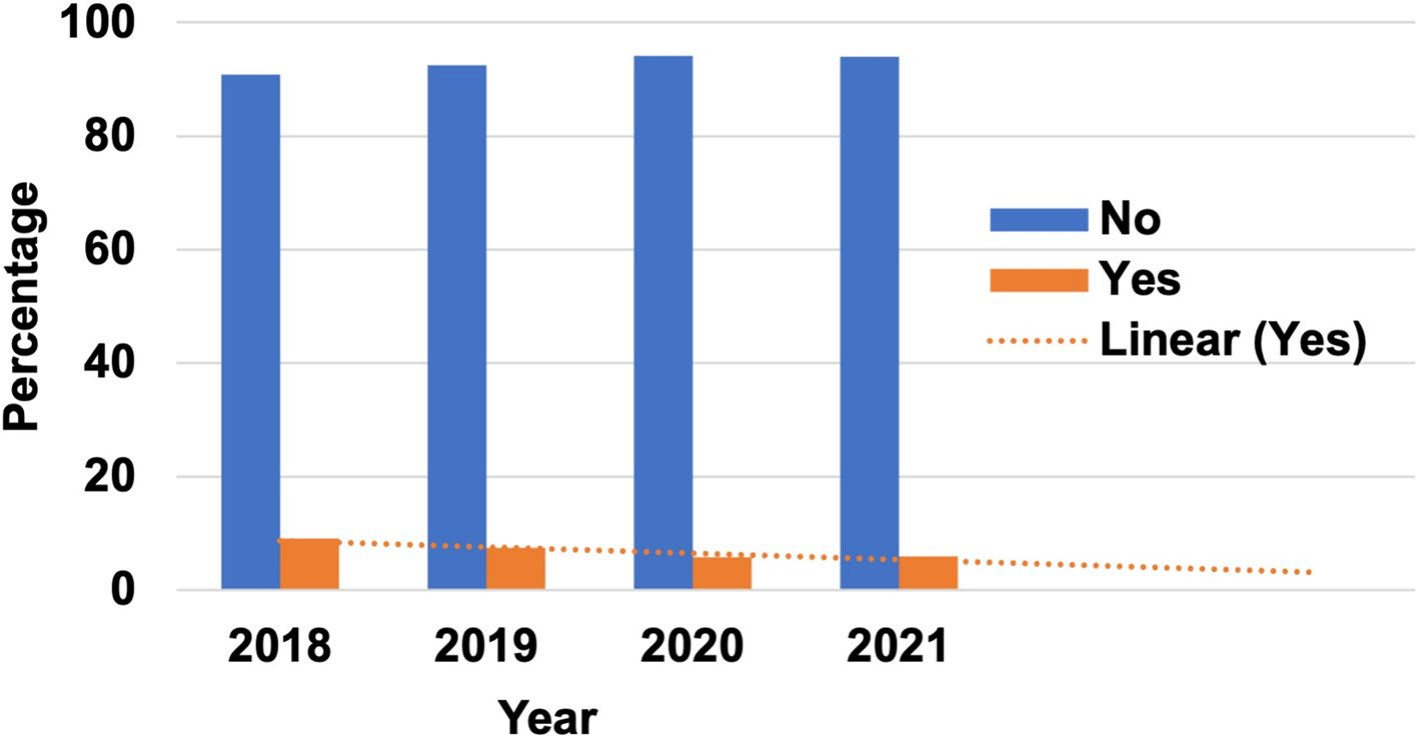
Trends in unmet healthcare needs by year. The dotted line represents the trendline for projected future unmet healthcare needs

The annual trends in the reasons for unmet healthcare needs are listed in Table 1. Throughout the study period, unmet healthcare needs decreased from 9.1% in 2018 to 5.8% in 2020 and increased slightly to 6.0% by 2021(p for trend: *p* < 0.001). The variation in unmet healthcare needs over four years was identified; the projected trend line for future unmet healthcare needs is presented in Fig. 1.

**Table 1:** Annual trends and causes of unmet healthcare needs

Variable	2018		2019		2020		2021	
	**N**	**%**	**N**	**%**	**N**	**%**	**N**	**%**
***Total***	6,121	100.0	5,527	100.0	5,259	100.0	5,024	100.0
***Unmet healthcare needs***								
No	5,561	90.9	5,114	92.5	4,952	94.2	4,741	94.0
Yes	560	**9.1**	413	**7.5**	307	**5.8**	283	**6.0**
***Reason for unmet healthcare needs***								
Lack of money	392	**70.0**	286	69.2	202	65.8	172	**60.8**
Difficulty moving	69	**12.3**	48	11.6	45	14.7	49	**17.3**
Lack of professional workforce related to the disabled	24	**4.3**	23	5.6	15	4.9	22	**7.8**
Previous bad experiences	16	2.9	13	3.1	12	3.9	11	3.9
Other	59	10.5	43	10.4	33	10.7	29	10.2

The percentage of those who stated a lack of money as a reason for unmet needs dropped significantly from 70% in 2018 to 60.8% in 2021. Difficulty in moving initially decreased in 2019 but increased again by 17.3% during the COVID-19 pandemic. In addition, the shortage of professional personnel in the disabled sector nearly doubled between 2018 and COVID-19.

### Baseline characteristics of the study population (2018, *N* = 6,121)

The baseline characteristics of the study population are shown in Table 2. Of the participants, 55.4% were men, 60.5% were over 50, and 54.5% were householders. Facial or internal organ disabilities were predominant, accounting for 56% of all disabilities, followed by mental and intellectual disabilities (18.1%), visual, hearing, and speech disabilities (11.6%), and multiple disabilities (4.6%). A total of 53.0% of participants had mild severity, and the average disability-related limitation of daily living score was 1.8. Among the participants, 26.8% required more than 30 min to reach a medical institution, 59.1% had depression, 53.7% reported being unhealthy, and 53.4% had a chronic disease.

**Table 2:** Baseline characteristics of the study population (2018, *N* = 6,121)

Variable/Category	Total		Unmet healthcare needs					*p*-value
			**Yes**		**No**		**x**^**2**^**/t**	
	**N**	**%**	**N**	**%**	**N**	**%**		
**Total**	6,121	100.0	560	9.1	5,561	90.9		
***Predisposing factors***								
** Sex**							0.02	0.897
Male	3,389	55.4	312	9.2	3,077	90.8		
Female	2,732	44.6	248	9.1	2,484	90.9		
Age group*							29.94	< 0.001
Under 20	1,047	17.1	61	5.8	986	94.2		
20–39	674	11.0	41	6.1	633	93.9		
40–59	2,350	38.4	245	10.4	2,105	89.6		
Over 60	2,050	33.5	213	10.4	1,837	33.5		
** Disability type**							23.19	0.002
Physical disabilities	950	15.5	105	11.1	845	88.9		
Brain lesions	875	14.3	86	9.8	789	90.2		
Visual disabilities	709	11.6	89	12.6	620	87.4		
Auditory/Linguistic	1,015	16.6	75	7.4	940	92.6		
Intellectual/Autism Spectrum Disorder	523	8.5	42	8.0	481	92.0		
Mental health disabilities	316	5.2	24	7.6	292	92.4		
Facial/Internal organ	1,312	21.4	109	8.3	1,203	91.7		
Multiple	421	6.9	30	7.1	391	92.9		
** Disability Severity**							5.38	0.020
Severe	3,242	53.0	270	8.3	2,972	91.7		
Mild	2,879	47.0	290	10.1	2,589	89.9		
***Enabling factors***								
** Householder**							26.90	< 0.001
Yes	3,006	49.1	334	11.1	2,672	88.9		
No	3,115	50.9	226	7.3	2,889	92.7		
** Marital status**							7.36	0.007
Yes	2,787	45.5	224	8.0	2,563	92.0		
No	3,334	54.5	336	10.1	2,998	89.9		
** Education status**							3.19	0.364
Elementary school or below	1,974	32.2	190	9.6	1,784	90.4		
Middle school	1,027	16.8	104	10.1	923	89.9		
High school	2,197	35.9	188	8.6	2,009	91.4		
College or above	923	15.1	78	8.5	845	91.5		
** Monthly household income(KRW **^**a**^**: ₩1,000)**							91.12	< 0.001
Under 1,000	1,351	22.1	202	15.0	1,149	85.0		
1,000–2,000	1,354	22.1	141	10.4	1,213	89.6		
2,000–3,000	1,133	18.5	82	7.2	1,051	92.8		
3,000–4,000	923	15.1	56	6.1	867	93.9		
Over 4,000	1,360	22.2	79	5.8	1,281	94.2		
** Disability-related† limitation in ADL**^**b**^** (Min:1, Max:4)**	1.81	0.62	1.95	0.62	1.79	0.62	5.68	< 0.001
** Travel time to health facilities**							12.39	0.004
Below 30 min	4,478	73.2	374	8.4	4,104	91.6		
Over 30 min	1,643	26.8	186	11.3	1,457	88.7		
***Need factors***								
** Depression**							42.53	< 0.001
Yes	3,620	59.1	404	11.2	3,216	88.8		
No	2,501	40.9	156	6.2	2,345	93.8		
** Self-rated health**							70.88	< 0.001
Good	2,833	46.3	164	5.8	2,669	94.2		
Poor	3,288	53.7	396	12.0	2,892	88.0		
** Chronic diseases**							67.87	< 0.001
Absent	2,855	46.6	168	5.9	2,687	94.1		
Present	3,266	53.4	392	12.0	2,874	88.0		

^**†**^Mean

^a^South Korea Won

^b^Activities of Daily Living

^*^Participants under 15 years of age may have had the questionnaire completed by a proxy respondent (the mother, father, or guardian)

Table 2 shows the differences between predisposing, enabling, and need factors for unmet and met healthcare needs. Householders (11.1%) had more unmet healthcare needs among the predisposing factors, but no variations regarding sex or education level were identified. Participants 40 or older had higher rates of unmet healthcare needs (40–59 years, 38.4%; > 60 years, 33.5%). Participants with visual (12.6%) and mild disabilities (10.1%) experienced more unmet healthcare needs. Participants with visual (12.6%) and mild disabilities (10.1%) experienced more unmet healthcare needs. Regarding enabling factors, participants with a “low” income level (15.5%) experienced more unmet healthcare needs than those with high-income levels. Disability-related limitations in ADL scores were higher in participants who experienced unmet healthcare needs. The participants who lived far from medical institutions (11.3%) experienced increased unmet healthcare needs. Among the need factors, participants who reported poor health (12.0%), chronic diseases (12.0%), and depression (11.2%) experienced increased unmet healthcare needs.

### Unmet healthcare needs among Korean people with disabilities (2018–2021)

The unmet healthcare needs for people with disabilities were significantly higher among those aged 40 to 59 years (aOR = 1.28, 95% CI = 1.10–1.50) compared to other age groups. According to disability types, those with visual (aOR = 1.36, 95% CI = 1.07–1.73) and intellectual or Autism Spectrum Disorder (ASD) (aOR = 1.52, 95% CI = 1.09–2.11) were more likely not to have access to healthcare services than other types. Regarding severity, the less severe the disability (aOR = 1.22, 95% CI = 1.05–1.41), unmet healthcare needs indicated a higher likelihood among householders (aOR = 1.22, 95% CI = 1.02–1.46) and without a spouse (aOR = 1.22, 95% CI = 1.03–1.43). The greater the disability-related limitation in ADL (aOR = 1.38, 95% CI = 1.24–1.53), and the longer the (aOR = 1.44, 95% CI = 1.27–1.63) travel time to health facilities. Unmet healthcare needs decreased as household income increased, showing an inverse relationship between income level and unmet healthcare needs. Compared to households earning less than 1 million KRW, those with an income of 2–3 million KRW (aOR = 0.63, 95% CI = 0.51–0.77), 3–4 million KRW (aOR = 0.57, 95% CI = 0.45–0.72), and over 4 million KRW (aOR = 0.58, 95% CI = 0.46–0.73) had significantly lower odds of experiencing unmet healthcare needs. Regarding self-reported factors, among people with disabilities, those with depression (aOR = 1.67, 95% CI = 1.45–1.92), poor self-rated health (aOR = 1.57, 95% CI = 1.38–1.79), and present chronic diseases (aOR = 1.90, 95% CI = 1.65–2.18) were more likely to not use healthcare services (Table 3).

**Table 3:** Unmet healthcare needs in Korean people with disabilities (2018–2021)

Variable	aOR ^a^	95% CI ^b^		*p*-value
***Predisposing factors***				
**Sex**				
Female	1.00			
Male	1.04	0.90	1.21	0.5619
**Age group**				
Under 20	1.00			
20–39	1.08	0.78	1.48	0.6474
40–59	0.99	0.72	1.35	0.9341
Over 60	1.28	1.10	1.50	0.0019
**Disability type**				
Physical disabilities	1.00			
Brain lesions	0.81	0.64	1.03	0.0911
Visual disabilities	1.36	1.07	1.73	0.0109
Auditory/Linguistic	0.91	0.72	1.16	0.4509
Intellectual/Autism Spectrum Disorder	1.52	1.09	2.11	0.0132
Mental health disabilities	0.84	0.58	1.21	0.3407
Facial/Internal organ	0.86	0.69	1.08	0.1946
Multiple	0.83	0.62	1.10	0.1978
**Severity of disabilities**				
Severe	1.00			
Mild	1.22	1.05	1.41	0.0110
***Enabling factors***				
**Householder**				
No	1.00			
Yes	1.22	1.02	1.46	0.0292
**Marital status**				
Yes	1.00			
No	1.22	1.03	1.43	0.0193
**Education status**				
College or above	1.00			
High school	1.04	0.84	1.28	0.7353
Middle school	1.09	0.85	1.39	0.4958
Elementary school or below	1.22	0.95	1.55	0.1202
**Monthly household income (KRW c: ₩1,000)**				
Under 1,000	1.00			
1,000–2,000	0.91	0.76	1.08	0.2619
2,000–3,000	0.63	0.51	0.77	<.0001
3,000–4,000	0.57	0.45	0.72	<.0001
Over 4,000	0.58	0.46	0.73	<.0001
** Disability-related limitations in ADL **^**d**^	1.38	1.24	1.53	<.0001
**Travel time to health facilities**				
Below 30 min	1.00			
Over 30 min	1.44	1.27	1.63	<.0001
***Need factors***				
**Depression**				
No	1.00			
Yes	1.67	1.45	1.92	<.0001
**Self-rated health**				
Good	1.00			
Poor	1.57	1.38	1.79	<.0001
**Chronic diseases**				
Absent	1.00			
Present	1.90	1.65	2.18	<.0001
**Year**				
2018	1.00			
2019	0.87	0.77	0.99	0.0298
2020	0.71	0.62	0.81	<.0001
2021	0.73	0.63	0.85	<.0001

^a^Adjusted Odds Ratio

^b^Confidence Interval

^c^South Korea Won

^d^Activities of Daily Living

^*^ Models are adjusted for sex, age group, householder, spouse, education status, disability type, severity of disabilities, monthly household income, disability-related limitations in ADL, travel time to health facilities, depression, self-rated health, chronic diseases, year

^**^QIC: 10,397.3260

## Discussion

This study aimed to identify the factors influencing the unmet healthcare needs of Korean people with disabilities and their underlying causes. This study focused on Andersen's Behavioral Model for vulnerable populations, with selected predisposing, enabling, and need factors as covariates [12, 13].

Our findings indicate that between 2020 and 2021, the number of people with disabilities with unmet healthcare needs decreased from 7.5% to 5.8%. In contrast, the number of people with disabilities with difficulty moving increased to 17.3%. The COVID-19 pandemic may have influenced these changes. During the COVID-19 pandemic, disparities in access to technology and the broadband Internet have reduced the availability of accessible public transportation [23] and home care by nurses and other health professionals [24, 25]. The lack of real-time communication about COVID-19 information and resources in accessible formats may have contributed to disparities in delayed healthcare and unmet needs for health services among people with disabilities. The baseline results revealed that people with mild disabilities, men, aged 40–59 years, those traveling over 30 min to health facilities, and those belonging to lower-income groups had a higher percentage of unmet healthcare needs. Consistent with previous findings, economic and social circumstances were positively associated with unmet healthcare needs [26, 27].

Our primary findings exhibited that “lack of money” was the most reported unmet healthcare needs barrier, followed by “difficulty in moving” in 2021. The percentage of those who experienced unmet needs due to a lack of money significantly decreased from 70% to 60.8%. The third most reported barrier to accessing healthcare services was a “lack of professional support for people with disabilities.” The lack of professional support for people with disabilities nearly doubled during the COVID-19 pandemic compared with 2018 figures. Park et al. [28] also suggested that transportation barriers impede the participation of individuals with severe physical and communication impairments in population screening for chronic diseases. Previous studies have also frequently cited transportation problems as barriers to accessing healthcare services for people with disabilities [29, 30].

We found that people with disabilities experienced unmet healthcare needs primarily due to a lack of money, difficulty moving, and a lack of a professional workforce in the disabled sector. Meanwhile, according to other studies, people without disabilities experience unmet healthcare needs mainly due to a lack of time and mild symptoms [31]. We found the people with disabilities aged 40–59 years highly unmet the healthcare needs. It was consistent in studies that reported, People with disabled adults aged 20–64 reported more than three times as many unmet health care needs [32, 33]. In contrast, people with disabilities, despite being covered by health insurance, often experience unmet healthcare needs owing to the financial burden of out-of-pocket expenditures [31]. Some studies in Iran indicate that PWD to promote their health need services that is not covered by health insurances, which led financial burden [34, 35]. In a relatively short period, South Korea has achieved universal healthcare coverage for its entire population, with the scope of health insurance gradually expanding. However, the rise in non-covered medical services led to a 62%–63% health insurance coverage rate in 2018 [36]. Limited health insurance coverage often results in high out-of-pocket costs for medical expenses, which can be a major factor contributing to unmet healthcare needs [37].

Disparities in access to healthcare caused by the COVID-19 pandemic may be closely linked to the implementation of public health measures such as self-quarantine, isolation, and social distancing, which disrupt service provision for people with disabilities [38] “Previous bad experiences” were also reported as barriers to accessing services. This finding highlights the importance of focusing on the satisfaction of people with disabilities [39]. Dissatisfied clients report higher levels of distress over time and often avoid seeking help when necessary because of past negative experiences [39].

Second, those with visual and intellectual disabilities experience more unmet healthcare needs than those with other disability types. Thus, a disability type gap exists among people with disabilities. Spencer et al. reported that transportation problems and service refusal by healthcare providers were the most significant barriers for people aged over 40 years [40]. Difficulties using stairs in an unfamiliar environment, walking on paths with broken blind tracks, and exiting a bus without readily available schedule information discourage visually impaired individuals from visiting a healthcare provider [41–44].

The experiences of unmet healthcare needs among individuals with intellectual disabilities also provide significant insights. This may be due to large differences in the healthcare quality experienced by people with intellectual disabilities, including sensory experience, communication, anxiety, access and advocacy, and systems issuesM [45]. Similarly, studies examining the healthcare experiences of adults with intellectual disabilities have revealed their dissatisfaction with healthcare providers [46]. In particular, adults diagnosed with ASD reported dissatisfaction with patient-provider communication [39, 47]. Additionally, people diagnosed with ASD reported several barriers to accessing and using health services, including difficulties in finding help, navigating the healthcare system, and describing their needs [39]. Children and adults diagnosed with ASD have more significant unmet healthcare needs when receiving mental health care and other specialized care [48]. The results of the present study support this conclusion. These findings underscore the importance of addressing specific healthcare barriers and tailoring support services to meet the diverse needs of people with disabilities in South Korea. Future research is warranted to investigate whether and how barriers to service utilization clustered by disability type can shed light on the health service experiences of people with disabilities.

This study had several limitations that should be considered when interpreting the findings. First, the sample included only people with disabilities identified by the Ministry of Health and Welfare from 2015 to 2017. Therefore, the data may not fully represent the entire population of people with disabilities in South Korea. Nevertheless, considering the high disability registration rate in South Korea and the methodological rigor in sample design, the potential impact of this limitation on the study results is expected to be minimal. Additionally, the database used in this study, managed by the Ministry of Health and Welfare, is the first comprehensive panel survey providing reliable statistics on registered individuals with disabilities, offering valuable insights into healthcare accessibility for this population [49]. Second, the DLDP relies on self-reported data rather than medical records, which may introduce recall bias and affect the reliability of survey responses. Third, the assessment of unmet healthcare needs is relatively simple, focusing primarily on hospital care. Furthermore, the proportion of individuals reporting unmet healthcare needs is relatively low compared to findings from other studies and settings, requiring cautious interpretation. To gain a deeper understanding of the various factors affecting healthcare accessibility, future research should consider employing qualitative approaches for further analysis. Fourth, due to dataset limitations, the classification system is based on predefined categories, which may not fully capture the diversity of all disability types [16]. Additionally, the DLDP classification differs from internationally recognized frameworks, such as the Washington Group Short Set. Since different classification criteria are used to define disability types, direct comparisons across studies should be interpreted with caution. Finally, a major limitation of this study is the absence of data on individuals without disabilities. Because the dataset does not include a non-disabled comparison group, it is not possible to assess the extent of disparities in unmet healthcare needs between people with and without disabilities. Future studies should consider incorporating comparative analyses with non-disabled populations to provide a broader perspective on healthcare accessibility disparities.

## Conclusions

This study is one of the first attempts to determine the trends and reasons for unmet healthcare needs among Koreans with disabilities by disability type using the 2018–2021 DLDP. Our study highlights that the most significant barriers to unmet healthcare needs for people with disabilities were economic constraints, difficulty moving, and travel times of more than 30 min to health facilities. This study contributes to national policy measures to address the unmet healthcare needs of Koreans with disabilities, as evidenced by the most recent national DLDP survey (2018–2021). This study suggests that people with disabilities must receive individualized social attention and support to address unmet healthcare needs based on their vulnerable characteristics.

## Supplementary Information

Supplementary Material 1.

## Data Availability

All data were available from the database of Korea’s Disabled People’s Development Institute (KODDI) (https://survey-koddi.gallup.co.kr/). KODDI controls access to these data and grants access to any researcher who follows the established ethical approval process outlined by KODDI.

## Abbreviations

aOR: Adjusted Odds Ratio
DLDP: Disability and Life Dynamics Panel
KDPDI: Korea Disabled People’s Development Institute
KRW: Korean won
ADLs: Activities of Daily Living
K-ADL: Korean Activities of Daily Living
CESD-11-D: Center for Epidemiologic Studies Depression Scale
GEE: generalized estimating equation
CI: confidence intervals
COVID-19: coronavirus disease 2019
ASD: Autism Spectrum Disorder
KODDI: Korea’s Disabled People’s Development Institute
