# Supplementary material for: Unmet healthcare needs of people with disabilities: evidence from the 2018–2021 Korean disability and life dynamics panel
Source: BMC Public Health. 2025 Jul 2;25:2263. doi: 10.1186/s12889-025-23048-w (PMC12219268; doi:10.1186/s12889-025-23048-w)
Supplement: Supplementary file 1 — Supplementary Material 1. [file 12889_2025_23048_MOESM1_ESM.docx]

| Supplementary table 1. Classification of Disability Types in South Korea | | | |
| --- | --- | --- | --- |
| **Major Categories** | **Middle Categories**  **(7 Types)** | **Detailed Categories (15Types)** | **Definition** |
| Physical Disability | Physical Disabilities | Physical Disability | Amputation, joint disorders, motor function impairment, spinal disorders, deformities |
|  | Brain Lesions | Brain Lesions | Complex disorders due to brain injury |
|  | Visual Disabilities | Visual Disability | Vision impairment, visual field defects, low vision (partial blindness) |
|  | Auditory/Linguistic | Auditory Disability | Hearing loss, balance function impairment |
|  |  | Linguistic Disability | Speech impairment, voice disorders, articulation disorders |
|  | Facial/Internal Organ | Facial Disability | Abnormalities due to atrophy, paralysis, or deformities of the facial region |
|  |  | Kidney Disorders | Cases requiring dialysis or kidney transplantation |
|  |  | Heart Disorders | Chronic or severe heart function impairment that restricts daily life |
|  |  | Liver Disorders | Chronic or severe liver function impairment that restricts daily life |
|  |  | Respiratory Organs Disorders | Chronic or severe pulmonary function impairment that restricts daily life |
|  |  | Gastrointestinal/Urological Disorders | Chronic or severe bowel or urinary disorders restricting daily life |
|  |  | Neurological Disorders | Chronic or severe brain function impairment restricting daily life |
| Intellectual or mental health disability | Intellectual/Autism Spectrum Disorder | Intellectual Disability | IQ of 70 or below |
|  |  | Autistim Spectrum Disorders | Autism spectrum disorder in children and adolescents |
|  | Mental health disability | Mental health disabilities | Schizophrenia, schizoaffective disorder, bipolar disorder, recurrent depressive disorder, psychiatric disorders due to brain organic damage, obsessive-compulsive disorder, epilepsy |
